# Ninth International Medical Congress

**Published:** 1887-10

**Authors:** Wm. Abram Love


### Society Reports.

### NINTH INTERNATIONAL MEDICAL CONGRESS.

### PROCEEDINGS OF GENERAL SESSIONS.

In the very outset of an effort to comply with your request to give you *a résumé* of the proceedings of the *Ninth International Medical Congress*, I meet with many difficulties, not the least of which was my want of ubiquity. This I must remedy by availing myself of the labors of others, and I will therefore, in the very outset, acknowledge my indebtedness to the editorial corps of *The Medical Register*, of Philadelphia (for the week published daily here), *The Medical Record*, of New York city, and the local press of the city of Washington, for to these I must of necessity be indebted for the facts furnished in great part for the compilation and completion of this communication.

### OPENING OF THE CONGRESS.

For the purpose of obtaining an eligible seat, I repaired to Albaugh's Opera House fully an hour before the time appointed for the opening. The house was already half filled, and a steady stream was pouring in which soon filled it to its utmost capacity. Soon the capacious stage presented a lively scene. Led by Dr. H. H. Smith, chairman of the Executive Committee; Dr. J. B. Hamilton, Secretary-General; Dr. A. Y. P. Garnett, chairman of the Committee of Arrangements, and Dr. N. S. Davis, the venerable founder and father of the American Medical Association, there came President Cleveland and Secretary Bayard; then followed fast the representatives from Merry Old England and her great metropolis; from Germany and France; from Ireland and

Scotland; from Austria and Switzerland; from Italy and Egypt, China and Japan; from Copenhagen, Ottawa and the East Indies; from Antwerp and Hague; from Halifax, Havana and Honolulu, all interspersed with representatives from the various States of the Union.

The appearance of President Cleveland was greeted with a cheer that made the Opera House ring to the center. At the appointed hour, 11 o'clock, Dr. H. H. Smith, chairman of the Executive Committee, arose and introduced "Grover Cleveland, President of the United States." He was greeted with prolonged cheers. When quiet was restored, he congratulated the country upon having so many of our own countrymen present, and upon having so many distinguished visitors from abroad who had gained eminence in their profession, and showed by their presence that they were devoted to the science of medicine in its further progress. He stated that his duty there was very pleasant and very brief. It was simply to declare the Ninth International Medical Congress open for organization and for the transaction of business.

Dr. H. H. Smith then proceeded to present the names of the officers for the Congress put in nomination by the Committee on Organization. The name of Dr. Nathan S. Davis, of Chicago, was presented for President. By acclamation he was elected unanimously, and at once entered upon the duties of his office. Next Dr. J. B. Hamilton, Surgeon-General of the United States Marine Hospital Service, was elected Secretary-General. Then followed a long list of Vice-Presidents, representing this and the foreign countries alluded to above, from one to many from each country, among whom were many of the leading medical minds of the world.

Next in order were elected the following Associate Secretaries: Wm. B. Atkinson, M. D., Philadelphia, Pa.; Geo. Byrd Harrison, M. D., Washington, D. C.; Frank Banga, M. D., Chicago, Ill.

Treasurer—E. S. F. Arnold, M. D., Newport, R. I.

Chairman Finance Committee—Richard J. Dunglison, M. D., Philadelphia, Pa.

Chairman Executive Committee—H. H. Smith, M. D., Philadelphia, Pa.

Chairman Committee of Arrangements—A. Y. P. Garnett, M. D., Washington, D. C.

Then came the election or confirmation of the Presidents of the several sections, numbering from I. to XVIII. inclusive. Then, too, was confirmed the appointment of the Secretaries, Vice-Presidents and Council members, which will be given, so far as space will permit, when reporting to you of the several sections.

The Congress being thus fully organized, the Secretary-General made a brief report, commencing with the invitation extended by the American Medical Association, May, 1884, to the Congress to meet in Copenhagen in the following August, to hold its next session in the United States. He then alludes to the acceptance of the invitation, the selection of Washington, and the work of the Committee of Invitation in completing the preliminary organization before the meeting of the American Medical Association at New Orleans in May, 1885, to the dissatisfaction there, the appointment of additional committeemen, the withdrawal of the original experienced members, and the difficulties in the way of carrying out, in all its details, the organization. He closed with compliments to the zeal and energy of Dr. H. H. Smith and the other members of the Executive Committee, whose labors have been crowned with the success shown in the organization just now completed.

At the conclusion of his report, Hon. Thos. F. Bayard, Secretary of State of the United States, was introduced to the Congress, and in a brief, but very appropriate and characteristic address, welcomed the foreign delegation to this country.

The cordial welcome extended by Secretary of State Bayard was responded to:

For England and Ireland, by Dr. W. H. Lloyd, of the Royal Navy.

For France, by Dr. Leon LeFort, of Paris.

For Germany, by Professor Unna, of Hamburg.

For Italy, by Senator Semmola, of Naples.

For Russia, by Professor Reyer, St. Petersburg.

At the conclusion of this ceremony, Dr. L. A. Sayre, of New York, was requested to take the chair, and the President-elect, Dr. N. S. Davis, delivered his address.

Dr. Garnett, chairman of the Committee of Arrangements, announced the following social programme:

*Monday Night*—General reception at Pension Hall.

*Tuesday Night*—Reception by the President at the White House. Members invited to visit the Corcoran Art Gallery.

*Wednesday, 4 o'clock p. m.*—Lawn party at ex-Commissioner Dent's.

*Thursday Night*—Banquet at Pension Hall to delegates and their ladies.

*Friday Evening*—Entertainment at Secretary Whitney's.

Members of the Congress requested to wear their badges during the entire session.

The Congress adjourned.

SECOND DAY—TUESDAY, SEPTEMBER 6, 1887.

The Congress met and at 10 o'clock was called to order by the President.

Dr. Austin Flint, of New York, occupied the hour on Fever, its cause and treatment.

He holds the position that fevers originate from micro-organisms, and that they are self-limited. Though he does not refer to the fact, still he takes the side of John Brown, of Edinburgh, in his fight with his master, Cullen, and of Todd, of London, in advocacy of the use of alcoholic stimulants in the treatment of fevers; advocates the use of anti-pyretics externally and internally, and warns against letting patients die from inanition. He regards essential fevers as defective nutrition, or the combustion and destruction of tissue, to save or prevent which the administration of hydro-carbons, alcoholic stimulants, would be a rational treatment, inasmuch as the oxidation of alcohol and the formation of water would serve to arrest the destruction of tissue and restore normal functional action.

The paper of Dr. Flint was ably gotten up and ably presented.

To be appreciated properly it should be taken as a whole. Any effort at abridgment would be an injustice to the author.

The General Congress then adjourned.

THIRD DAY—WEDNESDAY, SEPTEMBER 7, 1887.

The Congress was called to order by the President, who made some announcements and then requested Dr. C. F. Durante, of Rome, Italy, a Vice-President, to occupy the chair.

Dr. Mariano Semmola, of Naples, Italy, spoke in French and delivered the general address on "Bacteriology and its Therapeutical Relations." The address was replete with interest, passing with pleasant allusions to ancient and mediæval medicine to the present system of experimental medicine, and claims that if it is to become a science, it must not leave the experimental method. He regards the discoveries in the field of bacteriology as a long leap forward in the right direction, bringing to our knowledge the microscopic world with its myriads of enemies to mankind that we take into our systems in the air we breathe and the water we drink. While the doctor strongly advocates the idea of the axiom, "*cessante causa, cessat et effectus*," he fears that the great error of the day is the tendency to mistake *effect for cause* in making bacteriology a key to all pathology—making microbes the *cause* when they are but the effects of disease. He holds that of biological chemistry and the blood conditions suited to the culture of these microbes, we as yet know little, and that until this field is more thoroughly understood, speculation will bring error and delay in this search after truth. Professor Semmola says that bacteriology may lead to fruitful fields in the future, but it yet remains to be demonstrated to what extent microbes cause disease. Outside of carbuncle and tuberculosis, experimental researches have given no satisfactory results. He closed with an admonition to the younger generation to prosecute the research, to move with measured steps, to interrogate, not torture, nature, and he trusted that scientific research would be re-echoed in this land of independence.

A vote of thanks, unanimous, was tendered Senator Semmola for his able and interesting address.

FOURTH DAY—THURSDAY, SEPTEMBER 8.

Congress called to order, the President, Dr. N. S. Davis in the chair.

Dr. A. Y. P. Garnett, chairman Committee of Arrangements, introduced some resolutions relating to the contemplated international celebration in 1892, at Washington, D. C., in honor of the 400th anniversary of the discovery of America by Christopher Columbus, and an exposition of the arts and industries of all nations, asking that the Congress favor and commend the movement.

These resolutions were seconded by Senator Semmola in a brief but pointed, patriotic speech, and adopted unanimously.

Dr. A. L. Gihon, of the United States Navy, offered a resolution authorizing the President to appoint a committee of one from each nationality represented to select a place for the tenth meeting of the International Medical Congress. Adopted.

The President then introduced Dr. August Martin, of Berlin, resigning the chair to him, who in turn introduced Prof. P. G. Unna, of Hamburg.

Dr. Unna, speaking in German, delivered the address of the day on "*Dermatology in Relation to General Medicine.*" Dr. Unna earnestly commended the study of dermatology to the general practitioner—claimed for it a place among the pure sciences, but said that it needed ardent workers and earnest investigators to make it take its merited rank. He traced diseases to external causes—constitutional conditions and parasitic implantations—showing the influence of race, age and sex, of external agencies of season, climate and country. He alluded to the experimental researches that had already been made in this field, and recommended that in their further prosecution they be confined to the human species as more likely to lead to the discovery of new and interesting facts. He concluded by expressing the hope that the United States would be the first to lead off in the changes he had suggested. Sir James Grant-Bey proposed a vote of thanks for Prof. Unna's valuable paper, which was agreed to unanimously.

FIFTH DAY—FRIDAY, SEPTEMBER 9.

Congress called to order by the President. It was formally announced that the special committee had selected Berlin as the place for the meeting of the *Tenth International Medical Congress* of 1890. The President then vacated the chair in favor of Dr. Chas. D. F. Phillips, of London, who introduced Dr. G. F. Blandford, also of London, who entertained the Congress with an interesting paper on "*The Treatment of the Recently Insane in Public Asylums and Private Houses*."

Dr. Blandford did not propose to dwell upon chronic cases, but more particularly upon recent cases of acute insanity. He pointed out very clearly many of the prognostic symptoms, as the inter-paroxysmal frequency of the pulse; the moist, red tongue; the disturbed condition of the mind after sleep induced by narcotics; the rapidity of the invasion of the disease; the nature of the cause, whether mental or physical; from operations or exhaustion; whether from over-stimulation, inducing *mania a potu* and the duration of the stimulation. All these, he holds, have an important bearing in deciding the duration of the disease. He depicts in pointed terms the moral and social effects of commitment to insane asylums, and its influence in after life upon the inmates of such institutions.

Dr. Blandford, while he acknowledges the necessity for assignment of many cases to asylums, much prefers in *mania transitoris*, *delirium tremens* and kindred cases, treating them outside the asylums. He designates another class of cases that should at once be sent to the asylums.

In conclusion, Dr. Blandford refers to the laws of England, Scotland and of this country bearing upon the disposition of the insane, and seems to deprecate the condition of things in his own country.

On motion of Dr. A. Cordes, of Geneva, seconded by Dr. Kretchnar, of Berne, a vote of thanks was tendered unanimously to Dr. Blandford, with the announcement that the hour of meeting was 9:30 to-morrow.

The Congress adjourned.

SIXTH DAY—SATURDAY, SEPTEMBER 10.

The Congress was called to order. President Davis in the chair.

The Secretary-General, Dr. Hamilton, read a succession of resolutions from several sections, which were adopted by the Congress.

I. From Section XV. Public and International Hygiene, endorsing a paper by Dr. W. C. Cook, of Nashville, Tenn., recommending:

1st. Establishing a chair of hygiene in every medical college.

2nd. A chair in the universities and high schools.

3rd. A similar chair in all public schools.

4th. The establishment of a museum and laboratory of hygiene by every State.

II. From Section XVI. Medical Climatology and Demography:

Recommending the establishment in every country a national department bureau or commission for the collection of vital statistics, etc.

III. From Section III. Military and Naval Surgery:

1st. "Recommending the Ninth International Medical Congress to take such action as will direct the attention of all nations to the importance of preventing by international law the employment of explosive bullets in warfare."

At this stage of the proceedings, Dr. Grailey Hewitt, of London, came to the front, and with a few well-timed expressions of his own feelings, proceeded to read a paper, prepared and placed in his hands by the foreign delegation, expressive of their appreciation of the courtesies and hospitalities extended to them by the President of the United States and the people of this country, public and private, during their stay.

Dr. Martin, of Berlin, speaking in German, and Dr. Landolt, of Paris, speaking in French, adding their own feelings, reiterated the sentiments expressed by Dr. Grailey Hewitt. Dr. Edmund Owen, of London, then climbed upon the stage and made a rousing, rattling speech, sparkling with wit and humor, paying an eloquent tribute to our country, our people, our President and particularly "*Our queen of grace and beauty.*" From the beginning to the end of his eloquent and well-timed speech, he was interrupted by prolonged cheers.

Dr. Hamilton, Secretary-General, then, from the Section on Dental and Oral Surgery, presented to Dr. Hunt a gold-headed cane. He then feelingly returned thanks to all for their uniform kindness and courtesy to him personally, with thanks for the aids and efforts that had made the Congress a success—not the least of which was due to our foreign brethren, who had braved the shafts of calumny and the dangers of the deep to be with us here.

The President, Dr. Davis, then declared the Ninth International Medical Congress adjourned *sine die.*

The work of the several sections was carried on regularly during the week and would furnish much of deep interest to the profession, but the length of this communication forbids any allusion to that work here.

WM. ABRAM LOVE.

